# Adjuvant treatment with monosialoganglioside may improve neurological outcomes in neonatal hypoxic–ischemic encephalopathy: A meta-analysis of randomized controlled trials

**DOI:** 10.1371/journal.pone.0183490

**Published:** 2017-08-23

**Authors:** Lei Sheng, Zhaohui Li

**Affiliations:** Department of Pediatrics, The First People's Hospital of Jining, Jining, Shandong Province, China; Boston Children's Hospital / Harvard Medical School, UNITED STATES

## Abstract

**Background:**

Ganglioside has a neuroprotective role in neonatal hypoxic–ischemic encephalopathy (HIE). This study aimed to evaluate the neurological outcomes of monosialoganglioside as adjuvant treatment for neonatal HIE by conducting a meta-analysis.

**Methods:**

A comprehensive literature search was made in the Pubmed, EMBASE, Cochrane Library, Wanfang, CNKI, VIP databases through October 2016. Randomized controlled trials comparing monosialoganglioside with the usual treatment for newborns having HIE deemed eligible. Weighted mean difference (WMD) and risk ratio (RR) with 95% confidence interval (CI) were calculated for continuous and dichotomous data, respectively.

**Results:**

Ten trials consisting of 787 neonates were included. Adjuvant treatment with monosialoganglioside significantly reduced major neurodevelopmental disabilities (RR = 0.35; 95% CI = 0.21–0.57), cerebral palsy (RR = 0.32; 95% CI = 0.12–0.87), mental retardation (RR = 0.31; 95% CI = 0.11–0.88) as well as improved the mental (WMD = 14.95; 95% CI = 7.44–22.46) and psychomotive (WMD = 13.40; 95% CI = 6.69–20.11) development index during the follow-up. Also, monosialoganglioside significantly improved Neonatal Behavioral Neurological Assessment scores (WMD = 2.91; 95% CI = 2.05–3.78) compared with the usual treatment. However, adverse effects associated with monosialoganglioside were poorly reported in the included trials.

**Conclusion:**

Adjuvant treatment with monosialoganglioside had beneficial effects in improving neurological outcomes in neonatal HIE. However, these findings should be interpreted with caution because of methodological flaws in the included trials. Furthermore, safety of monosialoganglioside use should also be further evaluated.

## Introduction

Neonatal hypoxic ischemic encephalopathy (HIE) is a common cause of brain damage secondary to perinatal asphyxia, affecting 1 to 8 per 1000 live full-term births [1]. Approximately 25%–30% of these suffering neonates developed permanent neurologic disabilities [2]. HIE can be classified into mild, moderate or severe in accordance with Sarnat’s criteria [3]. The majority of neonates with mild HIE are usually associated with normal outcomes [4]. Severe HIE consequently contributes to a higher risk of neonatal death as well as long-term neurologic disabilities, including cerebral palsy, mental retardation, learning disability, and epilepsy [5]. Therapeutic hypothermia has been considered as standard treatment for neonates with moderate to severe HIE but is only partially effective [6]. Currently, well–established effective therapies are lacking [7] and supportive medical therapies to maintain physiologic parameters remain the standard therapy. For high rates of neurologic morbidities caused by HIE, development of new therapeutic agents is needed for the management of neonatal HIE.

Gangliosides are sphingolipids located predominantly in the neuronal membranes [8]. Gangliosides perform key roles in maintaining membrane integrity and in regulating brain development [9]. Significant reduction in several gangliosides was observed after hypoxia–ischemia [10]. Experimental studies showed that monosialoganglioside can protect against neuronal apoptotic insults [11, 12] and attenuate brain damage [13]. These findings revealed that monosialoganglioside may act as a promising therapeutic option for neonatal HIE. Monosialoganglioside exhibited promising outcomes in the management of neonatal HIE in China [14, 15]. However, the effect of monosialoganglioside as an adjuvant therapy on neuronal outcomes in neonatal HIE remains under debate.

Considering that monosialoganglioside treatment is mainly used in China but considerably less often in Western countries, we therefore evaluate the neuroprotective effects of monosialoganglioside as an adjuvant therapy in neonatal HIE by conducting a meta-analysis of randomized controlled trials (RCTs) in the Chinese literature.

## Materials and methods

### Search strategy

This meta-analysis was performed in adherence to the Preferred Reporting Items for Systematic Reviews and Meta-analyses Statement (S1 Table) [16]. PubMed, Embase, and Cochrane Library, WanFang, VIP, and China National Knowledge Infrastructure databases were searched through October 2016 for RCTs that compared monosialoganglioside to controls in newborns with moderate or severe HIE.A combination of the following search terms and Medical Subject Headings was applied for each selected database: (monosialoganglioside OR ganglioside) AND (neonate OR newborn OR infant) AND (hypoxic-ischemic encephalopathy OR encephalopathies OR birth asphyxia) AND (randomized controlled trial OR RCT OR random) (S1 File). In addition, we manually searched the bibliographies of relevant study to identify any possible trial.

### Study selection

Inclusion criteria for considering studies for this meta-analysis: 1) types of studies: RTCs; 2) types of participants: neonates with encephalopathy caused by perinatal asphyxia; 3) types of interventions: monosialoganglioside plus usual therapy versus usual therapy alone. Supportive care and other treatments were identical between two groups; and 4) outcome measures: primary endpoints were the incidence of major neurodevelopmental disabilities, cerebral palsy, mental retardation, and epilepsy. The secondary endpoints were Neonatal Behavioral Neurological Assessment (NBNA) scores at the end of treatment as well as mental development index (MDI), and psychomotive development index (PDI) during the longest follow-up. Usual therapy included control of seizures, reduction of encephalic pressure, elimination of brainstem symptoms, maintainance of normal ventilation, blood glucose, blood-gas or organ blood perfusion and symptomatic treatment. Trials included newborns with major congenital and hereditary abnormalities, congenital viral infections or other possible overt causes of neonatal encephalopathy.

### Data extraction and quality assessment

Two investigators independently scanned the titles or abstracts and extracted data. A standardized form was used to extract the characteristics of the included trials. Extracted data included the first author’s name, year of publication, sample size, gender, type of HIE, monosialoganglioside intervention, intervention and follow-up duration, and outcome measures. The methodological quality of the included trials was evaluated using the risk of bias tool of the Cochrane Handbook for Systematic Reviews of Interventions, including random sequence generation, allocation concealment, blinding of participants, personnel and outcome assessors, incomplete outcome data, selective outcome reporting, and other sources of bias.

### Statistical methods

Dichotomous data were calculated by the risk ratio (RR) with 95% confidence interval (CI) and continuous data were expressed as weighted mean difference (WMD) with 95% CI. Heterogeneity across the included trials was assessed using the *I*^*2*^ test and Cochrane Q statistic. A *I*^*2*^-value exceeding 50% was considered to indicate significant heterogeneity. We selected a random effects model when statistical heterogeneity was present; otherwise, a fixed-effect model was adopted. Potential publication bias was explored using the Egger's linear regression test and Begg's rank correlation test when the number of trials included in the analysis was exceeded 10 trials [17]. Sensitivity analyses were conducted by sequentially omitting anyone study each time. Subgroup analyses were conducted by treatment duration (>14 days versus ≤14 days), follow-up duration (≥12 months versus <12 months) and type of HIE (moderate and severe versus all types). All analyses were conducted using STATA 12.0 software (STATA Corp LP, College Station, TX, USA).

## Results

### Description of included trials

**Fig 1:**
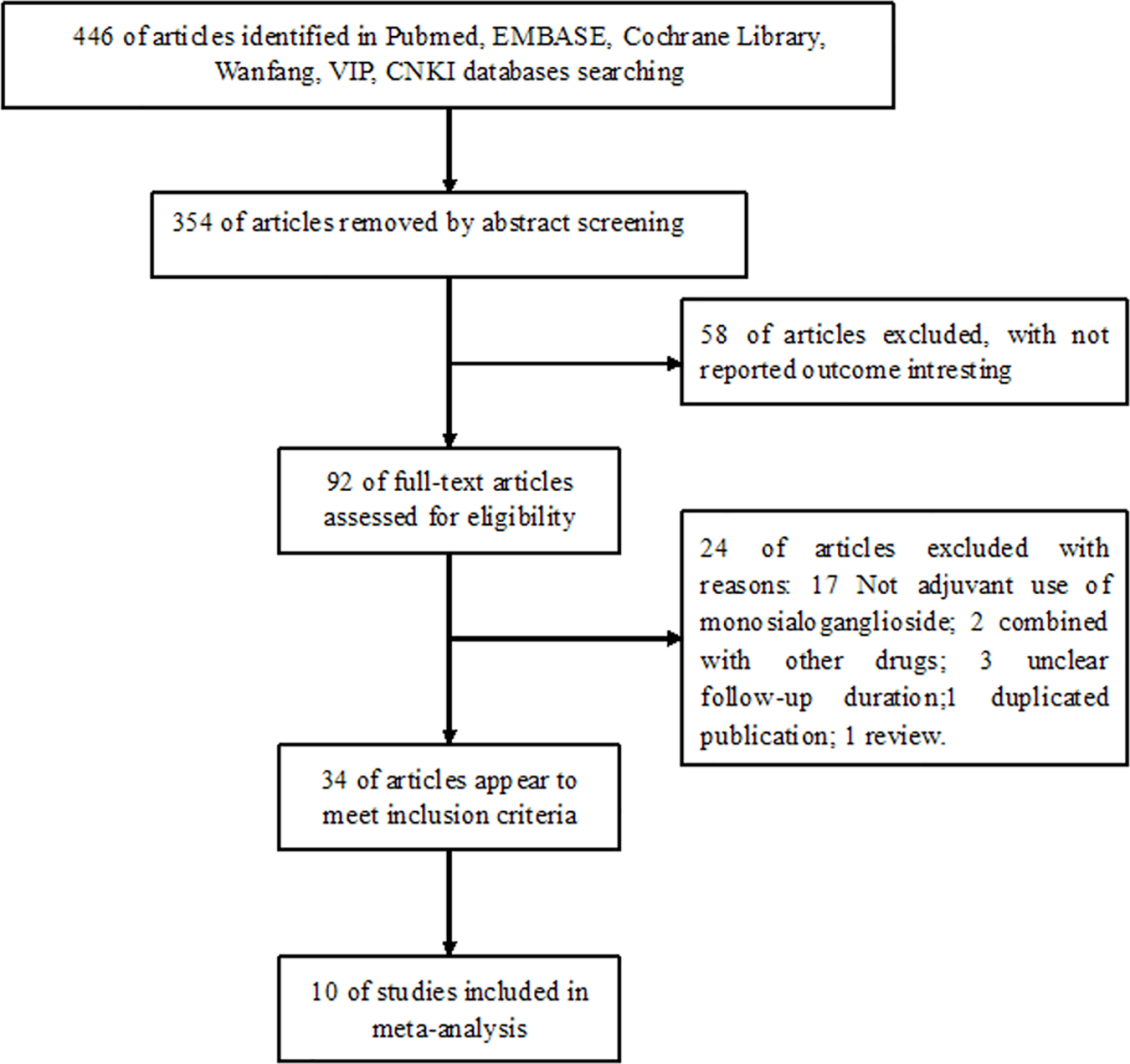
Flow chart of trial selection process.

Fig 1 presents the details of literature search and trial selection process. The PRISMA flow chart is shown in S1 Fig. In brief, a total of 446 potentially relevant publications were identified from the preliminary search. After the title and abstract were reviewed, 354 publications were excluded mainly due to being preclinical studies and reviews or lack of interesting outcomes. Among the 92 remaining records, 82 full-text articles were further removed after our predefined inclusion and exclusion criteria were applied. Thus, 10 RCTs [18–27] were finally included in the meta-analysis.

**Fig 2:**
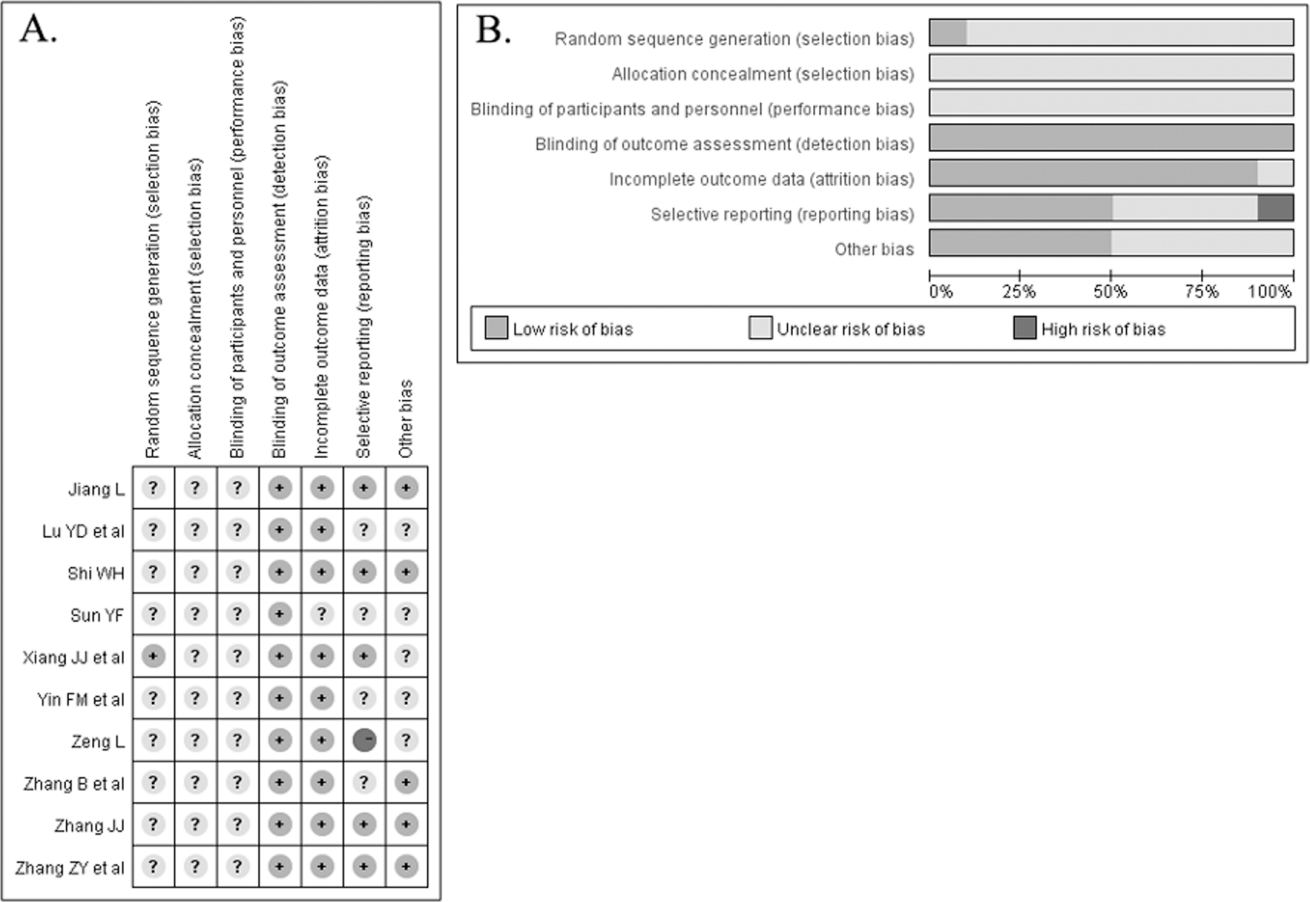
Risk of bias summary (A) and risk of bias graph (B)

The baseline characteristics of the included trials are summarized in Table 1. A total of 787 patients were identified and analyzed. All trials were published between 2005 and 2014 and conducted in China. Monosialoganglioside was administered at a dose of 20 g per day and treatment duration ranged from 7 days to 60 days. All included trials declared a randomized control design. The detailed methodological quality of the included trials is presented in Fig 2. Overall, most of the included trials were grouped as unclear risk of bias.

**Table 1:** Baseline characteristics of the trials included in the meta-analysis.

Study/Year	No. Patients	Male/female	Type of HIE			Main intervention		Treatment	Outcome	Follow-up
	(GM_1_/Con)	(GM_1_/Con)	Mild	Moderate	Severe	GM_1_ Group	Control group	Duration	Measures	(months)
Xiang JJ et al 2005 [15]	36/30	20/16 17/13	—	38	28	GM_1_ 20g/d, qd, IV drop + UT	UT	7–14 days	①+②+③+④+⑦	12 months
Lu YD et al 2008 [16]	44/42	29/15 28/14	—	58	28	GM_1_ 20g/d, qd, IV drop + UT	UT	20–28 days	⑤+⑥+⑦	12 months
Sun YF 2009 [17]	46/43	NP	—	NP	NP	GM_1_ 20g/d, qd, IV drop + UT	UT	20 days	①+⑦	6 months
Zeng L 2009 [18]	40/40	23/17 23/17	—	30	50	GM_1_ 20g/d, qd, IV drop + UT	UT	7 days	①	12 months
Zhang ZY et al 2009 [19]	43/40	21/22 18/22	42	33	8	GM_1_ 20g/d, qd, IV drop + citicoline + UT	Citicoline + citicoline + UT	20–30 days	①+⑤+⑥+⑦	6 months
Zhang B et al 2010 [20]	42/40	23/19 21/19	—	58	24	GM_1_ 20g/d, qd, IV drop + UT	UT	14 days	⑤+⑥+⑦	12 months
Yin FM et al 2010 [21]	31/30	18/13 17/13	—	34	27	GM_1_ 20g/d, qd, IV drop + UT	UT	20 days	⑤+⑥+⑦	4 months
Shi WH 2013 [22]	46/46	NP	—	42	50	GM_1_ 20g/d, qd, IV drop + UT	UT	30–60 days	②+③+④	15 months
Zhang JJ 2013 [23]	36/34	19/17 18/16	37	28	5	GM_1_ 20g/d, qd, IV drop + UT	UT	14 days	①+②+③	12 months
Jiang L 2014 [24]	39/39	21/18 22/17	17	33	28	GM_1_ 20g/d, qd, IV drop + citicoline + UT	Citicoline + citicoline + UT	14 days	①+②+③	12 months

Abbreviations: Con, control; NBNA, neonatal behavioral neurological assessment; HIE, hypoxic-ischemic encephalopathy; NP, not provided; UT, usual therapy; FDP, fructose-1,6-diphosphate GM_1_, monosialotetrahexosylganglioside; IV, intravenous injection. ①Major neurological disability; ②Cerebral palsy; ③Mental retardation; ④Epilepsy; ⑤Mental development index; ⑥Psychomotive development index; ⑦Neonatal behavioral neurological assessment.

### Neurodevelopmental disabilities

**Fig 3:**
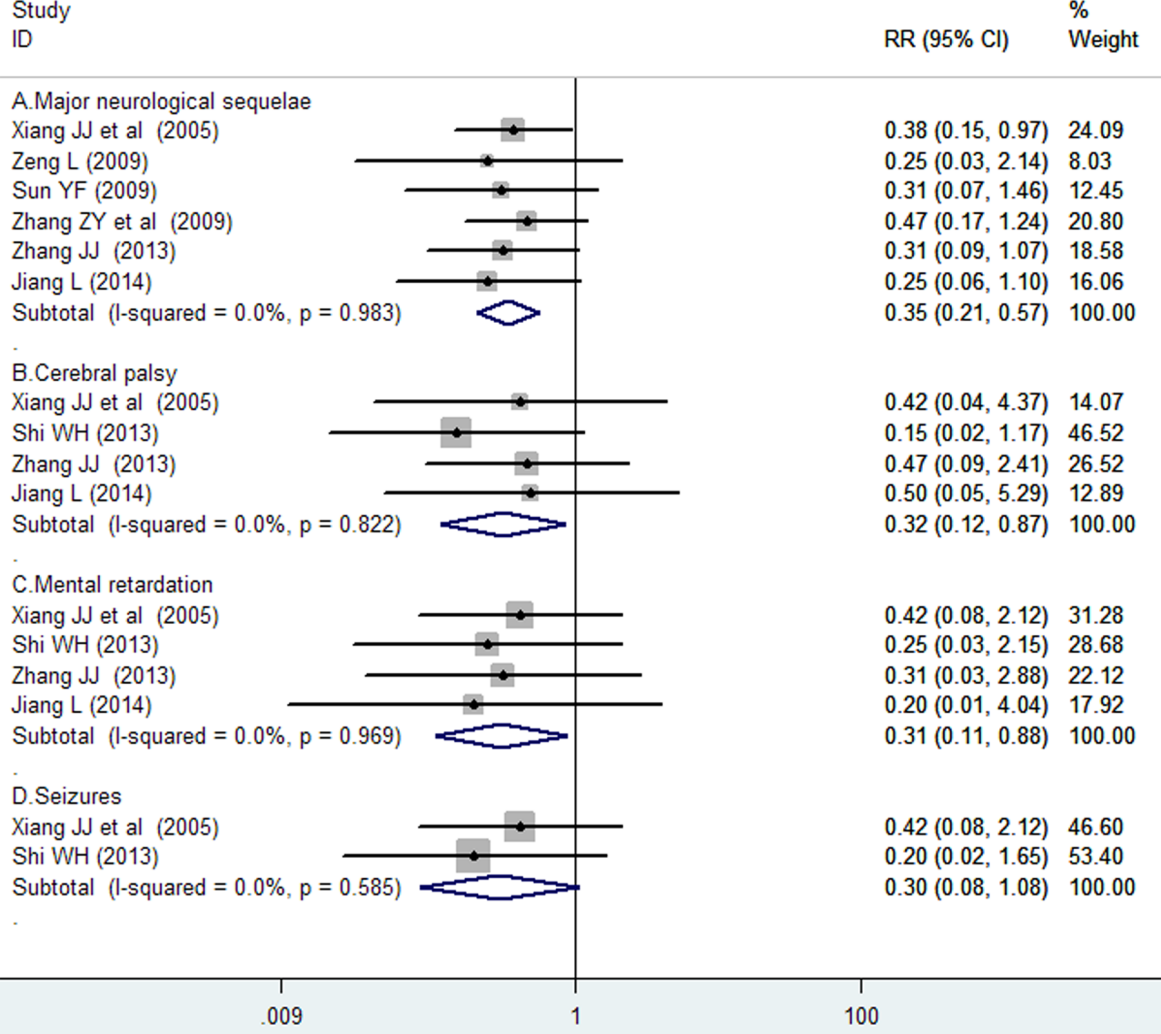
Forest plots showing risk ratio with 95% confidence interval of major neurodevelopmental disability comparing with or without monosialoganglioside treatment in a fixed-effect model.

Six trials [18, 20–22, 26, 27] reported data on major neurodevelopmental disabilities during follow-up. The number of major neurological disability in the monosialoganglioside and the usual therapy groups was 18 of 240 (7.5%) and 48 of 226 (21.2%), respectively. As shown in Fig 3A, monosialoganglioside treatment was associated with a significant reduction in the incidence of major neurodevelopmental disability compared with usual therapy care (RR = 0.35; 95% CI = 0.21–0.57) in a fixed-effect model, with no evidence of significant heterogeneity (*I*^*2*^ = 0%, p = 0.983). Subgroup analysis indicated that the effects of monosialoganglioside in reducing major neurodevelopmental disability were observed in each predefined subgroup (Table 2). Sensitivity analysis revealed that there were minimal changes in the pooling effect sizes with the omission of anyone trials.

**Table 2:** Subgroup analyses on major neurodevelopmental disabilities.

Subgroup	No. of trials	Pooled RR	95% CI	Heterogeneity between studies
Severity of HIE				
Moderate + severe	3	0.34	0.16–0.72	P = 0.931; I^2^ = 0%
All type	3	0.35	0.18–0.69	P = 0.762; I^2^ = 0%
Treatment duration				
>14 days	2	0.41	0.18–0.93	P = 0.667; I^2^ = 0%
≤14 days	4	0.31	0.17–0.60	P = 0.963; I^2^ = 0%
Follow-up duration				
<12 months	2	0.41	0.18–0.93	P = 0.667; I^2^ = 0%
≥12 months	4	0.31	0.17–0.60	P = 0.963; I^2^ = 0%

Abbreviations: HIE, hypoxic-ischemic encephalopathy; RR, risk ratio; CI, confidence interval.

Meta-analysis of four trials [18, 25–27] showed a beneficial effect of monosialoganglioside therapy in reducing the incidence of cerebral palsy (RR = 0.32; 95% CI = 0.12–0.87; Fig 3B) and mental retardation (RR = 0.31; 95% CI = 0.11–0.88; Fig 3C) in a fixed-effect model, respectively. However, meta-analysis of two trials showed no significant difference in the incidence of epilepsy (RR 0.30; 95% CI 0.08–1.08; Fig 3D) between the monosialoganglioside and control groups.

### Mental development index and psychomotive development index

**Fig 4:**
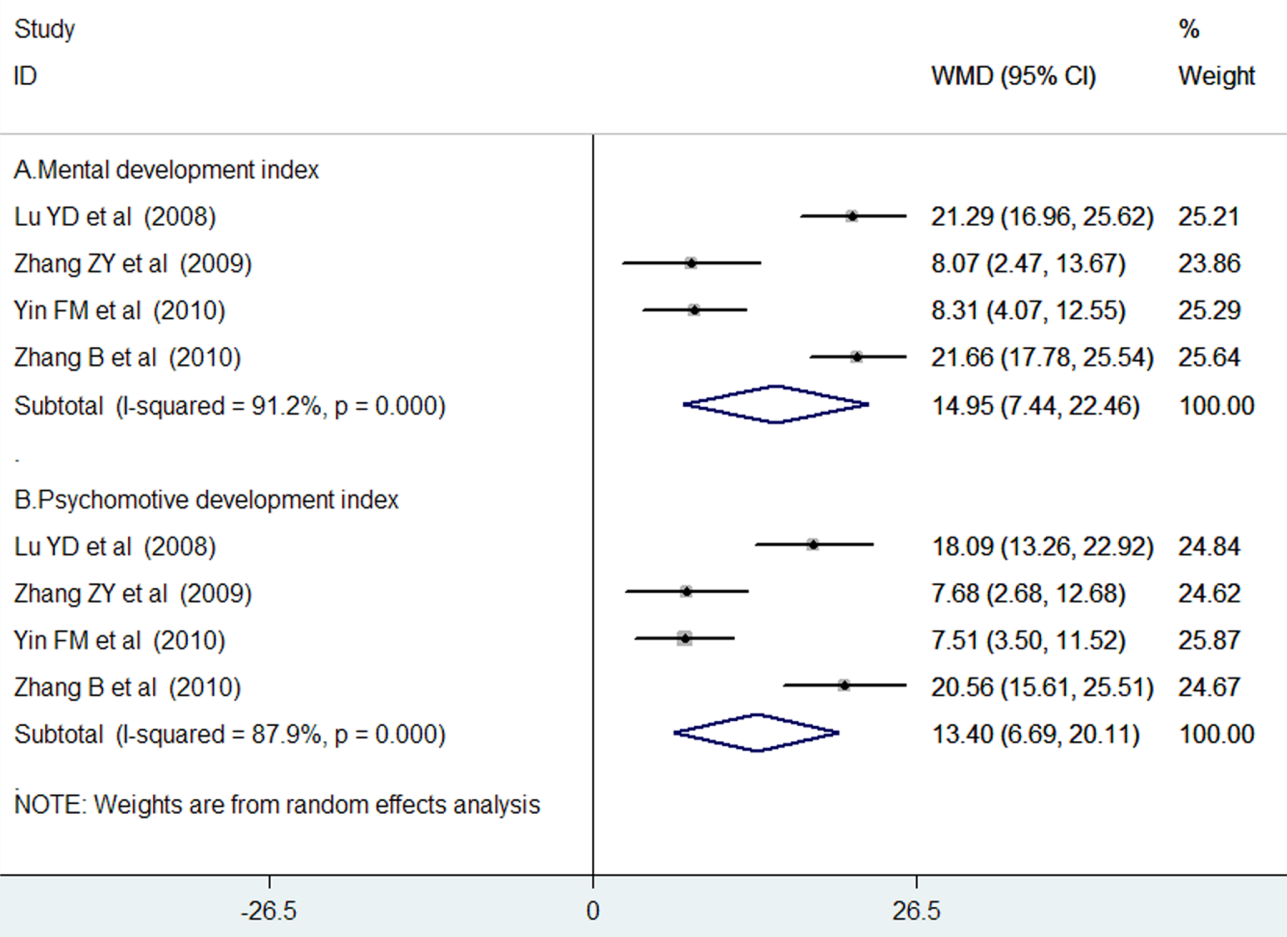
Forest plots showing weighted mean difference with 95% confidence interval of the Mental Development Index (A) and Psychomotive Development Index (B) comparing with or without monosialoganglioside treatment in a random effect model.

Four trials [19, 22–24] reported the data on MDI and PDI during the follow-up. As shown in Fig 4A, adjuvant treatment with monosialoganglioside resulted in a significant improvement in MDI (WMD = 14.95; 95% CI = 7.44–22.46) in a random effect model, with evidence of significant heterogeneity (*I*^*2*^ = 91.2%, p < 0.001). Similarly, adjuvant treatment with monosialoganglioside was associated with a significantly improvement in PDI (WMD = 13.40; 95% CI = 6.69–20.11; Fig 4B) in a random effect model, with evidence of significant heterogeneity (*I*^*2*^ = 87.9%, p < 0.001).

### Neonatal Behavioral Neurological Assessment

**Fig 5:**
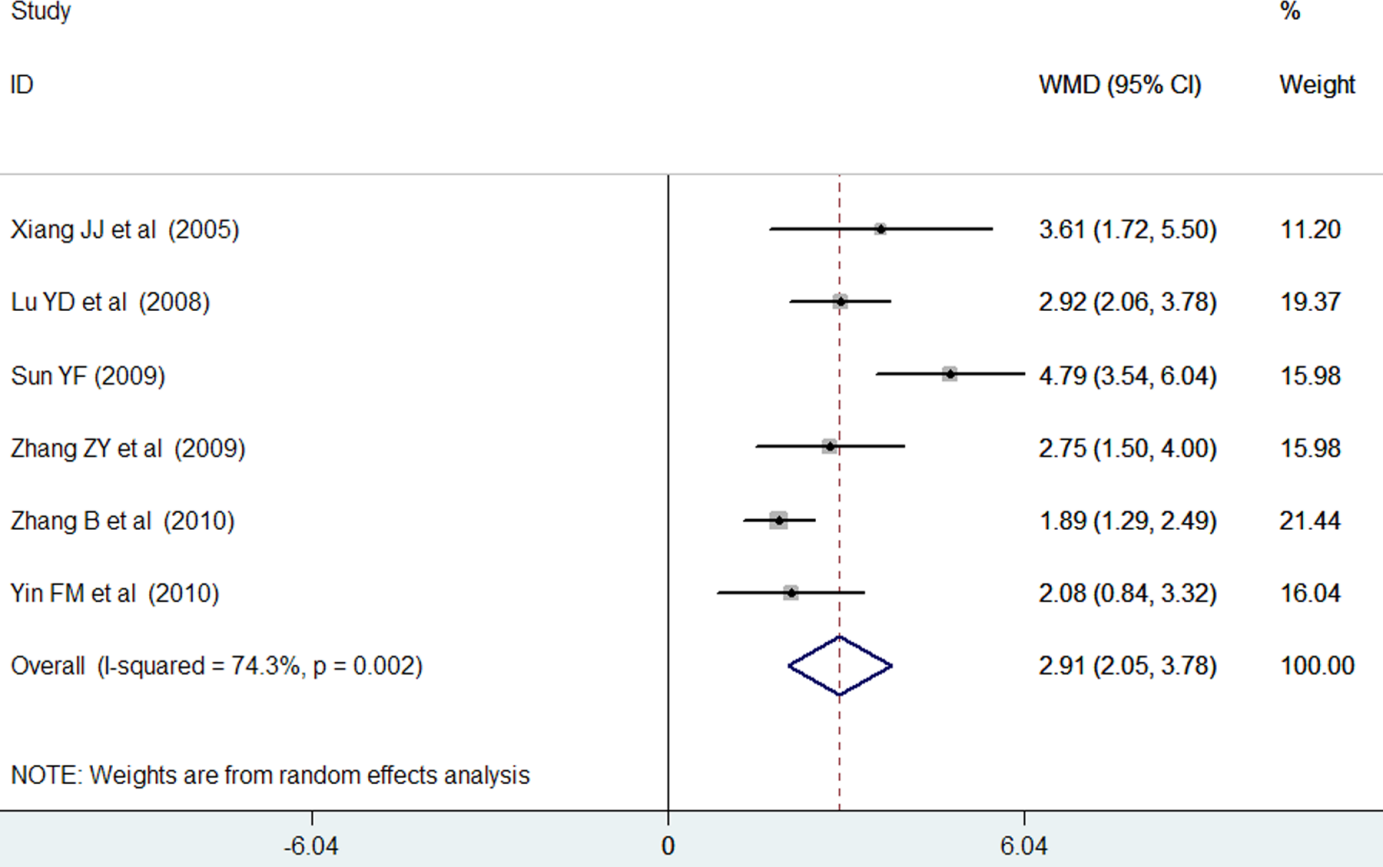
Forest plots showing weighted mean difference with 95% confidence interval of Neonatal Behavioral Neurological Assessment scores comparing with or without monosialoganglioside treatment in a random effect model.

Six trials [18–20, 22–24] reported the data on NBNA at the end of monosialoganglioside treatment. As shown in Fig 5, monosialoganglioside treatment was associated with a significant increment in NBNA scores (WMD = 2.91; 95% CI = 2.05–3.78) compared with the usual treatment in a random effect model, with evidence of significant heterogeneity (*I*^*2*^ = 74.3%, p = 0.002). Sensitivity analysis revealed that there were no change in the direction of effect sizes when we removed anyone trials in the overall analysis.

## Discussion

The main findings were as follows: 1) adjuvant treatment with monosialoganglioside appeared to reduce the risk of major neurodevelopmental disabilities including cerebral palsy and mental retardation during the follow-up; 2) increment of MDI and PDI scores were more evident after monosialoganglioside treatment compared with the usual therapy during follow-up; 3) monosialoganglioside treatment was associated with a higher NANB scores at the end of treatment. However, monosialoganglioside treatment appeared to exert no obvious effect on the development of epilepsy.

Neonates with mild HIE tend to not to show increased risk of neurological disabilities, whereas those survivors with severe HIE are at a high risk to develop major neurological disabilities [28, 29]. In the current study, the incidences of major neurodevelopmental disabilities in the monosialoganglioside and the usual therapy groups were 21.2% and 7.5%, respectively. Adjuvant treatment with monosialoganglioside significantly reduced the risk of major neurodevelopmental disabilities by 65%. Major neurological disabilities include ataxia, cerebral palsy, mental retardation, seizures or epilepsy. Monosialoganglioside also significantly reduced risk of cerebral palsy and mental retardation by 68% and 69%, respectively. However, did not evidently affect the reduction of the epilepsy rate. The negative finding may be correlated to lack statistical power because only two trials were included in the analysis.

The Bayley Scale of Infant Development including MDI and PDI was used to assess the motor and psychomotor development of neonates. MDI or PDI score <70 reflect severe developmental delay. NBNA was formulated according to the method of Bra-zelton and Amiel-Tison for behavioral neurological measurement in neonates [30]. Neurobehavioral abnormalities detected using the 20-item NABA reflect early manifestations of neurological status and may represent later neurodevelopmental disabilities. This meta-analysis indicated that adjuvant treatment with monosialoganglioside significantly improved the values of MDI, PDI and NANB compared with the usual therapy. These findings suggested that monosialoganglioside achieved additional short-term and long-term benefits in improving neurodevelopmental ability. In addition, adjuvant treatment with monosialoganglioside improved the recovery time of awareness, muscle tension, and primitive reflex of HIE newborns [26].

Despite the beneficial effects of monosialoganglioside in HIE newborns, one major concern is the potential adverse effects of the monosialoganglioside. However, adverse effects were poorly reported because the included trials did not select tolerance and safety as outcomes. The reported common adverse effects of monosialoganglioside included fever, chills, cyanosis, cold sweat, skin lesion, and even cardiovascular damages [31].Therefore, the safety of monosialoganglioside use should be further evaluated in future studies.

Several potential limitations should be indicated. First, most included trials lacked sufficient information on the randomization or allocation concealment method, therefore, the methodological flaws of the included trials were an important concern. Second, potential publication bias cannot be excluded because all included trials were published in Chinese. Finally, statistical heterogeneity was present in pooling continuous data. The differences in duration of regimens, follow-up periods, and severity of HIE across the included trials may partly contribute to the observed heterogeneity.

In conclusion, this meta-analysis indicates that adjuvant treatment with monosialoganglioside appears to offer additional benefits in terms of improving short-term clinical effects and reducing long-term neurodevelopmental disabilities. Given the methodological flaws of the included trials, these findings should be interpreted with caution. Determination of the optimal duration of intervention and long-time follow-up should be considered in future trials. Future studies should explore the underlying mechanisms of the protective roles of monosialoganglioside.

## Supporting information

S1 Table PRISMA checklist. (DOC)

S1 File Search strategy of each database. (DOC)

S1 Fig PRISMA flow chart. (DOC)
