# Supplementary material for: Adjuvant treatment with monosialoganglioside may improve neurological outcomes in neonatal hypoxic–ischemic encephalopathy: A meta-analysis of randomized controlled trials
Source: PLoS One. 2017 Aug 23;12(8):e0183490. doi: 10.1371/journal.pone.0183490 (PMC5568297; doi:10.1371/journal.pone.0183490)
Supplement: S1 File — (DOC) [file pone.0183490.s002.doc]

### S2 File Search strategy of each database.

Medical Databases---Pubmed and Embase

Limits: English Language, publications prior to October,31 2016, humans only

# 1 (“monosialoganglioside” [MeSH Terms] OR “ganglioside” [Free terms])

# 2 (“hypoxic-ischemic encephalopathy” [MeSH Terms] OR “encephalopathies” [Free Terms] OR “birth asphyxia” [Free Terms])

# 3 (“neonate” [MeSH Terms] OR “newborn” [Free Terms] OR “infant” [Free Terms])

# 4(“randomized controlled trials” [MeSH Terms] OR “RCTs” [Free Terms] OR “random” [Free Terms]

# 5 (#1) OR (#2) OR (#3) OR (#4).

Chinese Databases---China National Knowledge Infrastructure, VIP, and WanFang Data.

# 1 (“神经节苷脂[Title/Abstract] ” OR “施捷因” [Full text] OR “申捷” [Full text]

#2 (“缺氧缺血性脑病”[Full Text] OR “脑损伤”[Full Text] OR “窒息” [Full Text]

# 3 (“新生儿”[Full Text])

# 4 (“随机” [Full Text] OR “对照” [Full Text]

# 5 (#1) OR (#2) OR (#3) OR (#4).
