# Supplementary material for: Adjuvant treatment with monosialoganglioside may improve neurological outcomes in neonatal hypoxic–ischemic encephalopathy: A meta-analysis of randomized controlled trials
Source: PLoS One. 2017 Aug 23;12(8):e0183490. doi: 10.1371/journal.pone.0183490 (PMC5568297; doi:10.1371/journal.pone.0183490)
Supplement: S1 Fig — (DOC) [file pone.0183490.s003.doc]

**Identification**

**Screening**

**Eligibility**

**Included**

446 of articles identified in Pubmed, EMBASE, Cochrane Library, Wanfang, VIP, CNKI databases searching

354 of articles removed by abstract screening

92 of full-text articles assessed for eligibility

34 of articles appear to meet inclusion criteria

58 of articles excluded, with not reported outcome intresting

10 of studies included in meta-analysis

24 of articles excluded with reasons: 17 Not adjuvant use of monosialoganglioside; 2 combined with other drugs; 3 unclear follow-up duration;1 duplicated publication; 1 review.
